# Dynamic CT Perfusion Imaging of the Myocardium: A Technical Note on Improvement of Image Quality

**DOI:** 10.1371/journal.pone.0075263

**Published:** 2013-10-09

**Authors:** Daniela Muenzel, Sven Kabus, Bettina Gramer, Vivian Leber, Mani Vembar, Holger Schmitt, Moritz Wildgruber, Alexander A. Fingerle, Ernst J. Rummeny, Armin Huber, Peter B. Noël

**Affiliations:** 1 Department of Radiology, Klinikum rechts der Isar, Technische Universitaet Muenchen, Munich, Germany; 2 Philips Research Laboratories, Digital Imaging Department, Hamburg, Germany; 3 Philips Healthcare, CT Clinical Science, Cleveland, Ohio, United States of America; The University of Chicago, United States of America

## Abstract

**Objective:**

To improve image and diagnostic quality in dynamic CT myocardial perfusion imaging (MPI) by using motion compensation and a spatio-temporal filter.

**Methods:**

Dynamic CT MPI was performed using a 256-slice multidetector computed tomography scanner (MDCT). Data from two different patients–with and without myocardial perfusion defects–were evaluated to illustrate potential improvements for MPI (institutional review board approved). Three datasets for each patient were generated: (i) original data (ii) motion compensated data and (iii) motion compensated data with spatio-temporal filtering performed. In addition to the visual assessment of the tomographic slices, noise and contrast-to-noise-ratio (CNR) were measured for all data. Perfusion analysis was performed using time-density curves with regions-of-interest (ROI) placed in normal and hypoperfused myocardium. Precision in definition of normal and hypoperfused areas was determined in corresponding coloured perfusion maps.

**Results:**

The use of motion compensation followed by spatio-temporal filtering resulted in better alignment of the cardiac volumes over time leading to a more consistent perfusion quantification and improved detection of the extend of perfusion defects. Additionally image noise was reduced by 78.5%, with CNR improvements by a factor of 4.7. The average effective radiation dose estimate was 7.1±1.1 mSv.

**Conclusion:**

The use of motion compensation and spatio-temporal smoothing will result in improved quantification of dynamic CT MPI using a latest generation CT scanner.

## Introduction

Dynamic contrast enhanced CT scans enable collection of kinetic parameters (time-to-peak, mean-transit-time etc.). For instance, dynamic CT myocardial perfusion imaging (MPI) could help assess the hemodynamic significance of coronary artery stenosis on the associated myocardial tissue. In the last decade, coronary CT angiography (CCTA) has gained increased importance for non-invasive evaluation of the coronary arteries and the detection of coronary artery disease (CAD) [1]–[7]. For clinical practice the quantification of myocardial perfusion is of high relevance for diagnosis, prognosis and therapy of CAD. Initial studies illustrated MPI as a feasible and promising method for the assessment of myocardial perfusion deficits [8]–[11]. However, there are diagnostic limitations with respect to image quality and radiation exposure. Low tube output typically used in MPI to address the issue of radiation dose could result in relatively high image noise. In addition, movement of the heart during the scanning procedure could result in spatial mis-alignment of the targeted regions and reduce the accuracy of time- and space-dependent evaluation of myocardial perfusion in specific regions of interest (ROI), thus creating a need for motion correction methods.

The aim of this study was to assess the potential of MPI on a wide detector CT scanner in combination with motion correction and a spatio-temporal filter for improved quantification and reduced sampling resulting in radiation dose savings.

## Methods

### CT Myocardial Perfusion Examination

In this study data from two different patients – with and without myocardial perfusion defects – were evaluated to illustrate potential improvements for MPI. Institutional review board approval (Klinikum rechts der Isar, Technische Universitaet Muenchen) and written informed consent was obtained from all patients before enrollment in the study. Both patients with known CAD underwent magnetic resonance (MR) perfusion imaging and also invasive coronary angiography (ICA) within 4 weeks following perfusion imaging.

Patient 1 is a 57-year-old male with known coronary atherosclerosis and significant (>90%) stenosis of the left circumflex artery (LCA) and the intermedial branch with prior unsuccessful interventional recanalization. The distal RCA is functionally occluded. MR imaging showed perfusion deficits in the inferior, inferolateral and lateral wall of the left ventricle with accompanying hypokinesia of the left ventricular (LV) wall and a reduced ejection fraction. Patient 2 (80-year-old, male) has known CAD without interventional therapy in the past. No significant perfusion deficits were seen in MR perfusion imaging, and ICA showed coronary atherosclerosis but no significant coronary stenosis.

Dynamic MPI was performed using a 256-slice multidetector CT scanner (MDCT) scanner with 80 mm z-axis-coverage (Brilliance iCT, Philips Healthcare, Cleveland, OH, USA) at 80 kVp, 250 mAs, 0.27 sec gantry rotation time and 250 mm field-of-view (FOV), with 360° reconstructions of 3 mm thick slices using the CA (smooth) kernel. Data was acquired during pharmacologically induced stress (Adenoscan, Sanofi-Aventis Frankfurt, Germany; 140 µg/kg/min) in end-expiratory breath-hold. ECG-triggered axial scans with zero increment were performed at a specific cardiac location following an injection of 40 ml contrast medium (4 ml/s, Imeron 400 MCT, Bracco Imaging Deutschland GmbH, Konstanz, Germany) using a dual injector (Stellant, MEDRAD, Inc., Indianola, Pennsylvania, USA). Scans were triggered at alternate cardiac cycles at end-systole (40% of the RR interval), resulting in 15 acquisitions over a period of 30 cycles. On this note, the option of scanning every alternate cardiac cycle was chosen to reduce the radiation exposure to the patient.

### Motion Compensation

The aim of motion compensation is to spatially align the entire set of acquisitions such that all scans are in the same reference frame. Here, a two-stage approach is chosen: in the first stage, the motion between any two adjacent scans (time frames) is estimated resulting in a displacement vector for every single voxel. The second stage concatenates the displacement vectors from all time frames in order to transform each scan towards the designated scan [12].

Motion estimation between two scans is also known as an “image registration” task. For clarity we define a set of 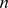 time frames by 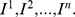 Given two adjacent frames 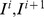 defined on an image domain 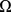 (as a subset of 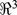) a registration algorithm aims at finding a displacement field 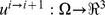 such that 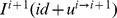 is most similar to 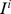. In mathematical terms, the similarity is described by a functional 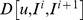, here chosen as the popular sum of squared differences (SSD),
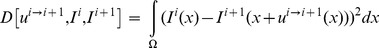
(1)


A registration based only on a similarity measure may yield a deformed template image which perfectly matches the reference image as long as all gray values are present in both images. However, the problem is ill-posed and the underlying deformation does in general may not be applicable in a physical context. Therefore, an additional smoothness constraint (or regularizer) is considered which can be chosen to model the application specific physical properties. It should be noted that the regularization term prevents the resulting vector field from an unwanted physiologically implausible behavior (e.g., strong local expansion or compression or even folding). In addition, by regularization the registration algorithm is less affected by image noise, imaging artifacts or abrupt inflow of contrast agent. In this note we employ a regularizer based on the popular linear elastic potential
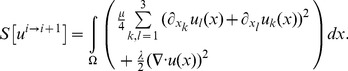
(2)


Its elastic properties are modeled by Lamé parameters 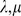, which can be translated into Young’s modulus (linked to tissue stiffness) and Poisson’s ratio (contraction perpendicular to applied stretch). Specifically, 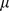 is inversely proportional to the elastic modulus and 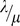 is proportional to the incompressibility of the material. To address the heart motion occurring during the scanning procedure on the one hand and the inflow of contrast agent on the other hand, a relatively large Young’s modulus (four times higher as for lung deformations) as well as a small Poisson’s ratio were chosen. As common to registration algorithms, absolute values from the literature cannot be used since their choice depends on the intensity scale of the acquired scans, on the chosen similarity measure as well as on implementation issues.

By combining the similarity measure and the regularizing term, the problem can be formulated as finding a displacement field 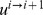 which minimizes the joint functional.
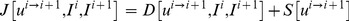
(3)


The computation of the Gâteaux derivative of (1) and (2) yields a necessary condition for 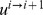
being a minimizer of (3). The outcome is a system of nonlinear partial differential equations,
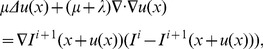
(4)equipped with associated boundary conditions.

For the discretization of (4) finite differences in conjunction with Neumann boundary conditions have been chosen. The resulting system of linear equations consists on one hand of a sparse, symmetric and highly structured matrix arising from the regularizer and, on the other hand, of a so-called force vector corresponding to the similarity measure (see [13] for further details). The system of equations is then linearized and iteratively solved by a conjugate gradient scheme. The iteration is stopped if the update in 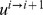 is below 0.05 mm for all positions indicating convergence.

To ensure convergence and to increase robustness, the algorithm was embedded into a multi-resolution scheme. In the interest of a fast clinical workflow, the original resolution level was skipped from processing. Runtime was measured in the range of about one minute.

### Spatial Filtering

An edge-preserving filter that reduces image noise in homogeneous regions while preserving the boundaries between different regions of tissue was used. Three-dimensional spatial smoothing was performed individually on each single time frame according to the method described by Isola et al [14].

### Temporal Filtering

Following spatial filtering and motion compensation, temporal smoothing was implemented as a 3-point weighted moving average filter, and applied individually to each pixel over time. The filter weights were chosen as (0.25, 0.5, 0.25). This filter configuration was chosen to ensure a satisfactory tradeoff between noise removal and preservation of the shape of the perfusion curves.

### CT Myocardial Perfusion Image Analysis

To emphasize possible improvements three datasets for each patient were generated: (i) original data, with no correction, (ii) motion compensated data, and (iii) motion compensated data with spatio-temporal filtering. All images were analyzed on a CT workstation (Brilliance Workspace [EBW], V4.5.2, Philips Healthcare, Cleveland, OH, USA) equipped with a prototype post-processing application (Dynamic Myocardial Perfusion). Image noise was calculated by the standard deviation of the mean CT attenuation values (HU) of the LV myocardium. Contrast-to-noise ratio (CNR) was calculated as mean HU within a specific ROI divided by image noise. Therefore, several ROIs of area 1 cm^2^ were manually placed in the hypoperfused areas. As a control, equally sized ROIs were defined in remote myocardium of the lateral wall of the left ventricular chamber and the interventricular septum. Precision in definition of normal and hypoperfused areas was evaluated by three experienced radiologists (range 5–7 years) in corresponding coloured perfusion maps for peak enhancement (PE). Using the 17 segment model recommended by ACC/AHA/ASNC [15], semi-quantitative visual assessment of myocardial perfusion (normal versus hypoperfusion) was performed. Kappa values were calculated to compare interobserver agreement on a per-patient basis for myocardial territories.

The effective dose of CT perfusion analysis was estimated by the product of the DLP (given by the scanner) and a conversion coefficient for the chest (k = 0.014 mSv*mGy^−1^*cm^−1^) [16].

## Results

### Image Noise and Contrast-to-noise-ratio

Image noise was 17.7 HU in standard images, 10.9 HU in motion-corrected images and 3.8 HU in filtered and motion-corrected images. Accordingly, CNR within a defined ROI was increased using motion correction and ST-filter with 31.6 versus 6.8 in standard reconstructions and 11.7 in motion-corrected images.

### CT Myocardial Perfusion Image Analysis

**Figure 1:**
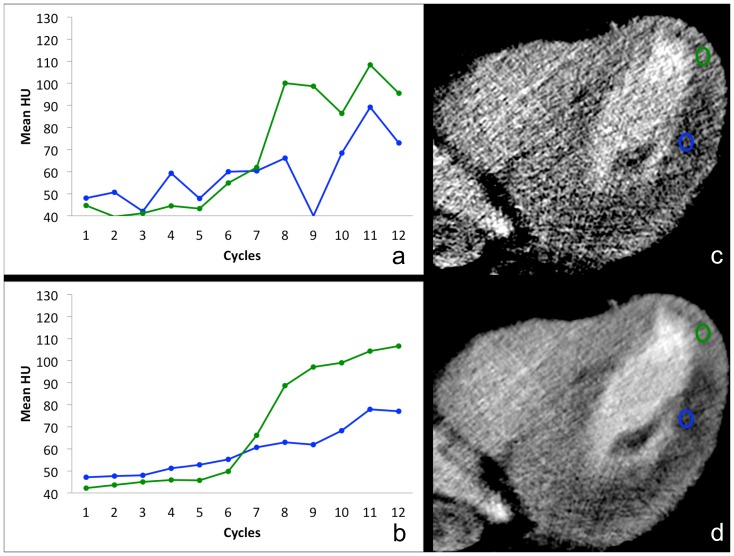
Time density curves of myocardial enhancement in Patient 1 before (a) and after motion correction and spatio-temporal smoothing (b). Hounsfield units (HU) of two regions-of-interest (ROI) are plotted against the time after contrast administration with a total of 12 scan cycles. The green curve represents the dynamic course of contrast enhancement of the normal apical LV myocardium, and the blue line illustrates delayed and decreased contrast enhancement of the hypoperfused myocardium of the inferolateral LV wall. Both curves are smoother with less variation after motion correction and spatio-temporal filtering (Fig. 1b). Figs. 1c and d show the axial images (original [1c] and motion corrected with spatio-temporal smoothing [1d]) with the corresponding ROIs – green for the normal region and blue in the region of decreased contrast enhancement. Both CT images are presented using the same window level and window width.

**Figure 2:**
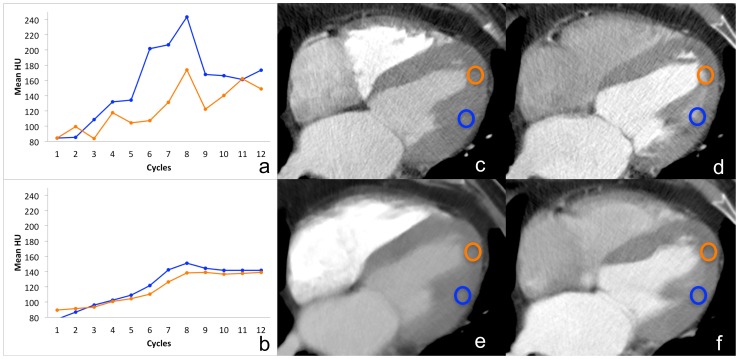
Dynamic contrast enhancement of the LV myocardium apical (orange) and inferolateral (blue) in Patient 2 with a HR varying from 60–110 bpm and several extrasytoles during CT perfusion examination. Perfusion analysis of original images shows irregular zigzag pattern of the resulting curves (a). A continuous run of the time density curves is illustrated after motion correction and spatio-temporal filtering (b). Both curves (orange and blue) illustrate normal contrast enhancement of the LV myocardium with no evidence of perfusion deficit. Figs. c-f: Shown are the axial images (and the ROIs) at two different instances in time – the original uncorrected (c, d) and after motion correction plus spatio-temporal smoothing (e, f). Images c and e are from cardiac cycle 3; d and f are from cardiac cycle 8. In the original images (c & d), the two ROIs do not cover the same area of the myocardium at both time points, but instead cover adjacent tissue and the contrast-filled lumen of the LV, resulting in several spikes in (a). In contrast, consistent measurements of the same part of the myocardium after motion correction (e & f) leading to coherent time-density curves (b) for both ROIs. All CT images are presented using the same window level and window width.

Figure 1 illustrates a representative example of Patient 1 with a large hypodense region in the inferior and inferolateral LV wall. Figure 1a shows the time attenuation plots of the mean CT numbers over time in the post-stenotic territory of the lateral LV wall (blue) and the remote myocardium (green) without the use of motion correction, with Figure 1b showing the corresponding plots after the use of motion correction and spatio-temporal smoothing. In addition, the CT images before and after motion correction and spatio-temporal smoothing are presented in Figures 1c & 1d, respectively. Similar curves are shown for Patient 2 (Figure 2), who had no significant perfusion deficits detected in CT or MRI perfusion analysis, and also no hemodynamically significant coronary stenosis found in interventional angiography performed in the catheter laboratory. In Patient 2, arrhythmia with multiple extrasystoles and a mean heart rate of 70 bpm (range 60–110 bpm) caused increased motion of the heart between the individual data acquisitions of the perfusion examination. Consequently, a defined ROI may not represent the same area of the myocardium during the course of time, but instead cover adjacent tissue or the contrast-filled lumen of the LV during different temporal instances (Figure 2c and d). This could help explain the spikes (false peaks) of 170 HU and 240 HU in Figure 2a. However, after motion correction, consistent measurements of the same part of the myocardium result in coherent time-density curves for both measurements in the apical and lateral wall. From Figures 1 and 2, it is clear that the use of motion correction and spatio-temporal smoothing makes it easier to quantify perfusion measurements, especially in smaller regions of interest where it is critical to spatially align structures of interest over time.

**Figure 3:**
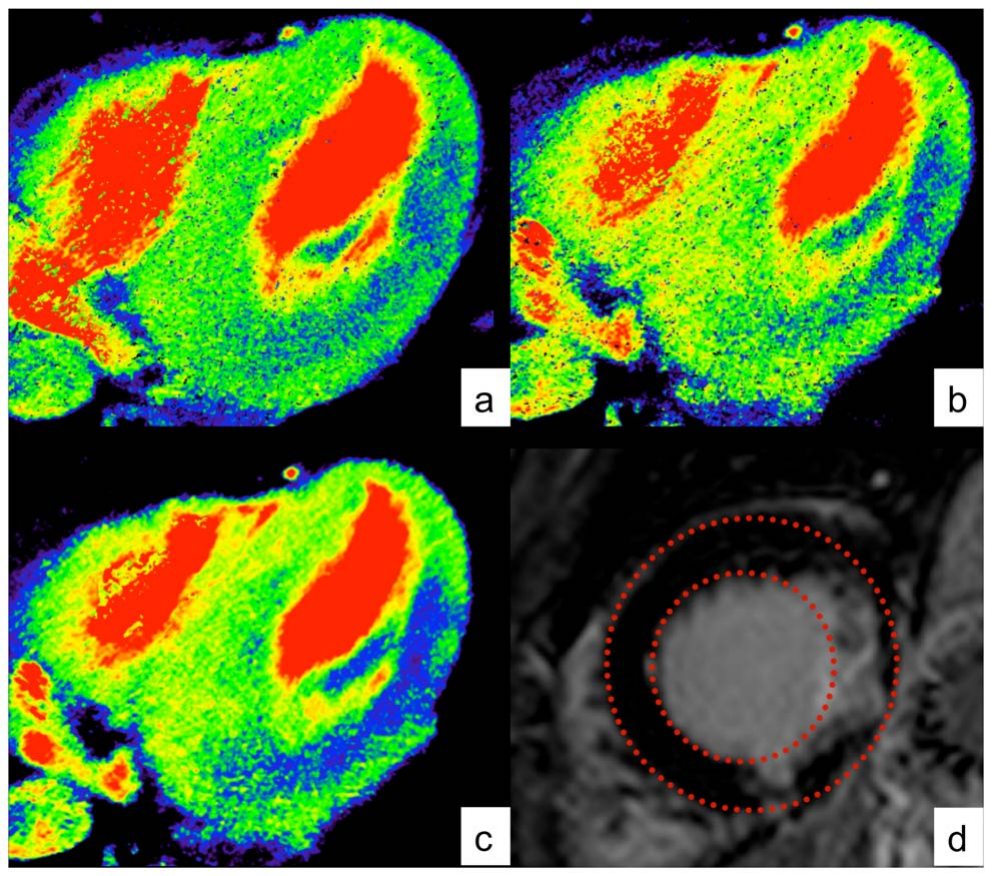
Coloured perfusion maps for original data (a), motion corrected data (b) and combined motion-corrected and spatio-temporal filtered data (c) of Patient 1 in axial orientation. As a reference, short axis view late enhancement MR images are shown (d) presenting partly transmural infarction of the inferolateral wall. LV endocardium and epicardium are outlined in red. The perfusion defect of the inferior and lateral LV wall is visible in all CT perfusion images, with improved delineation of the hypoperfused and remote myocardium using motion correction (b) and best image quality using combined reconstruction algorithm with motion correction and spatio-temporal filtering (c). Thus the transmural extent of the infarction as diagnosed in MRI (d) can be distinguished only in combined motion-corrected and spatio-temporal filtered images (c).

Figure 3 (a–c) shows the color maps for peak enhancement (PE) of Patient 1 in the axial images of the original data (3a), motion corrected data (3b) and combined motion-corrected and spatio-temporal filtered data (3c), demonstrating better delineation of the transit regions using both post-processing algorithms. Hypoperfusion of the myocardium of the lateral LV wall can be detected in all CT images 3a–c. Clearly, in addition to any incremental improvements brought about by the use of motion compensation (shown in Figure 3b), the additive use of spatio-temporal filter results in a better visualization of smaller transmural parts within the mostly hypoperfused area of the lateral LV wall as demonstrated in Figure 3c in contrast to the original color maps (3a). Figure 3d shows the corresponding MRI image (short axis view, late enhancement images) of Patient 1, confirming the myocardial hypoperfusion of the lateral LV wall with partly transmural extension. It is not possible to make the diagnosis whether there is a transmural extent of hypoperfusion in Figures 3a and b, but there is an excellent analogy of the MRI image (3d) and the color map using both motion compensation and spatio-temporal filtering (3c), illustrating a precise delineation of the transmural portion within the hypoperfused area. Accordingly, all three radiologists assessed transmurality of ischemia only in motion-compensated and filtered images. Interobserver agreement for segment evaluation (hypoperfusion versus normal) was very good with kappa values of 0.90, 0.95, and 0.95, respectively.

Mean DLP was 507±77 mGy*cm and mean CTDI_vol_ was 63±9 mGy, resulting in an average effective radiation dose in these case studies of 7.1±1.1 mSv.

## Discussion

Dynamic CT myocardial perfusion imaging is a feasible and promising technique for a quick and noninvasive assessment of myocardial perfusion and the detection of perfusion abnormalities. Several studies have shown promising results, offering the ability to evaluate the hemodynamic significance of coronary artery stenosis in CT imaging [8]–[11], [17], [18]. Currently, noninvasive assessment of the hemodynamic significance of coronary artery disease is mostly assessed by magnetic resonance imaging (MRI) and single photon emission computed tomography (SPECT) in clinical routine examinations of the heart. MRI is a well-established approach for the evaluation of myocardial perfusion deficits, and multiple studies proved a high diagnostic accuracy of MRI for the detection of CAD [19]–[21]. In addition, SPECT is the most widely used imaging modality for the determination of hemodynamic significant stenosis in CAD [22]. However, both techniques have limitations: MRI has a long procedure time, and patients with cardiac pacemakers or claustrophobia cannot be examined. SPECT requires injection of a gamma-emitting radionuclide (e.g. Tc-99m sestamibi or Tl-201) and exposes patients to a radiation dose in the range of 9–13 mSv or higher [23]. Therefore, an extended approach of CT-based analysis of anatomical and functional aspects of CAD is of special interest.

In this study, we presented options (post-processing algorithms) to improve the quality of dynamic CT of the myocardium in a reduced radiation dose setting. The principal findings of our study have been that the use of motion correction and spatio-temporal filter results in: (a) an enhanced image quality, (b) better differentiation between normal and hypoperfused areas and (c) improved diagnostic quality of perfusion examinations. Additionally, we limited the radiation exposure by scanning every other cardiac cycle.

For perfusion analysis, contrast enhancement for each voxel is assessed over a period of time. If the target organ (myocardium) shifts over time, standard perfusion evaluation tools cannot identify this discrepancy. As a result such tools are prone to including HUs of adjacent tissues that could appear because of spatial mis-alignment. Thus, the non-registered images suffer from artifacts caused by motion of the heart and motion of the whole chest e.g. due to breathing, and possible imperfect matching of the scan with the ECG interval. Those artifacts distort dynamic volume CT assessment of myocardial perfusion and may lead to misinterpretation of assessed values for perfusion measurements, for example in coloured perfusion maps.

In this preliminary study, we did not observe any loss of diagnostic information with the use of motion compensation and the spatio-temporal filter. Instead, the use of these post-processing algorithms actually reduced the blurring of boundaries between normal and small and hypodense regions of the myocardium, thereby improving the quality of perfusion maps. For instance, using these algorithms, we were able to better detect the extent of hypoperfused territory (endocardial versus transmural, Fig. 3), thus leading to a higher diagnostic confidence. Motion corrected and filtered images clearly depict the parts of transmural extent of myocardial infarction of the left lateral myocardial wall, as also shown in the late Gadolinium enhancement MR images. Differentiation of subendocardial versus transmural extent of infarction is of special clinical importance as the prognosis of restoration of myocardial contractile function depends on the degree of infarct transmurality [24]–[26]. Up to now, cardiac MR imaging including late enhancement has been established as a standard of reference for the assessment of transmurality of myocardial infarction [24]–[26]. In this feasibility study we could show that visualization of transmural infarction can be distinguished in CT after motion correction and temporal filtering, whereas in raw uncorrected CT data the evaluation of the transmurality of infarction was impaired.

With regard to z-coverage, compared to the previous generations of MDCT scanners, the increased detector width of 256 or more slices offers also for perfusion imaging the possibility to image the whole left ventricular myocardium without table movement. So the complete target volume can be imaged within each scanning circle, and there is no need of conjunction of adjacent scan volumes for assessing a complete perfusion scan of the heart.

This study was designed as a feasibility study for first evaluation of motion compensation and a spatio-temporal filter in CT MPI. Therefore, only two patients were included for clinical evaluation. This is a major limitation of our study, and these findings have to be proved in a clinical trial with a larger sample size.

We demonstrated that dynamic CT MPI is a fast and accurate tool for detection of perfusion abnormalities. With the proposed post-processing steps the quality of MPI increases and the accuracy for diagnostics increases. So this technique for the first time offers the possibility to differentiate between subendocardial and transmural infarction in patients with myocardial ischemia in CT imaging. With this in mind a future step could be to further optimize radiation dose to the patient while using the proposed post-processing tools in combination the modern reconstruction algorithms, such as iterative reconstruction techniques.

## References

[1] Earls JP, Berman EL, Urban BA, Curry CA, Lane JL, et al (2008) Prospectively gated transverse coronary CT angiography versus retrospectively gated helical technique: improved image quality and reduced radiation dose. Radiology 246: 742–753. 18195386 10.1148/radiol.2463070989

[2] Scheffel H, Alkadhi H, Leschka S, Plass A, Desbiolles L, et al (2008) Low-dose CT coronary angiography in the step-and-shoot mode: diagnostic performance. Heart 94: 1132–1137. 18519548 10.1136/hrt.2008.149971

[3] Hirai N, Horiguchi J, Fujioka C, Kiguchi M, Yamamoto H, et al (2008) Prospective versus retrospective ECG-gated 64-detector coronary CT angiography: assessment of image quality, stenosis, and radiation dose. Radiology 248: 424–430. 18574140 10.1148/radiol.2482071804

[4] Muenzel D, Noel PB, Dorn F, Dobritz M, Rummeny EJ, et al (2012) Coronary CT angiography in step-and-shoot technique with 256-slice CT: impact of the field of view on image quality, craniocaudal coverage, and radiation exposure. Eur J Radiol 81: 1562–1568. 21561732 10.1016/j.ejrad.2011.04.027

[5] Muenzel D, Noel PB, Dorn F, Dobritz M, Rummeny EJ, et al (2011) Step and shoot coronary CT angiography using 256-slice CT: effect of heart rate and heart rate variability on image quality. Eur Radiol 21: 2277–2284. 21710267 10.1007/s00330-011-2185-4

[6] Chao SP, Law WY, Kuo CJ, Hung HF, Cheng JJ, et al (2010) The diagnostic accuracy of 256-row computed tomographic angiography compared with invasive coronary angiography in patients with suspected coronary artery disease. Eur Heart J 31: 1916–1923. 20233790 10.1093/eurheartj/ehq072

[7] de Graaf FR, Schuijf JD, van Velzen JE, Kroft LJ, de Roos A, et al (2010) Diagnostic accuracy of 320-row multidetector computed tomography coronary angiography in the non-invasive evaluation of significant coronary artery disease. Eur Heart J. 31: 1908–1915. 10.1093/eurheartj/ehp571 20047991

[8] Bamberg F, Becker A, Schwarz F, Marcus RP, Greif M, et al (2011) Detection of hemodynamically significant coronary artery stenosis: incremental diagnostic value of dynamic CT-based myocardial perfusion imaging. Radiology 260: 689–698. 21846761 10.1148/radiol.11110638

[9] So A, Wisenberg G, Islam A, Amann J, Romano W, et al (2012) Non-invasive assessment of functionally relevant coronary artery stenoses with quantitative CT perfusion: preliminary clinical experiences. Eur Radiol 22: 39–50. 21938441 10.1007/s00330-011-2260-x

[10] Bastarrika G, Ramos-Duran L, Rosenblum MA, Kang DK, Rowe GW, et al (2010) Adenosine-stress dynamic myocardial CT perfusion imaging: initial clinical experience. Invest Radiol 45: 306–313. 20421800 10.1097/RLI.0b013e3181dfa2f2

[11] Blankstein R, Shturman LD, Rogers IS, Rocha-Filho JA, Okada DR, et al (2009) Adenosine-induced stress myocardial perfusion imaging using dual-source cardiac computed tomography. J Am Coll Cardio 54: 1072–1084. 10.1016/j.jacc.2009.06.014 19744616

[12] Kabus S, Lorenz C (2010) Fast elastic image registration. Proc. Medical Image Analysis For The Clinic–A Grand Challenge, MICCAI 81–89.

[13] Modersitzki J (2004) Numerical Methods for Image Registration. Oxford University Press, New York.

[14] Isola AA, Schmitt H, van Stevendaal U, Begemann PG, Coulon P, et al (2011) Image registration and analysis for quantitative myocardial perfusion: application to dynamic circular cardiac CT. Phys Med Biol 56: 5925–5947. 21860077 10.1088/0031-9155/56/18/010

[15] Cerqueira MD, Weissman NJ, Dilsizian V, Jacobs AK, Kaul S, et al (2002) Standardized myocardial segmentation and nomenclature for tomographic imaging of the heart: A statement for healthcare professionals from the Cardiac Imaging Committee of the Council on Clinical Cardiology of the American Heart Association. Circulation 105: 539–542. 11815441 10.1161/hc0402.102975

[16] McCollough C (2008) The measurement, reporting, and management of radiation dose in CT. College Park, MD: American Association of Physicists in Medicine AAPM report no.96.

[17] Rocha-Filho JA, Blankstein R, Shturman LD, Bezerra HG, Okada DR, et al (2010) Incremental value of adenosine-induced stress myocardial perfusion imaging with dual-source CT at cardiac CT angiography. Radiology 254: 410–419. 20093513 10.1148/radiol.09091014 PMC2809927

[18] Cury RC, Magalhães TA, Borges AC, Shiozaki AA, Lemos PA, et al (2010) Dipyridamole stress and rest myocardial perfusion by 64-detector row computed tomography in patients with suspected coronary artery disease. Am J Cardiol 106: 310–315. 20643238 10.1016/j.amjcard.2010.03.025

[19] Cury RC, Cattani CA, Gabure LA, Racy DJ, de Gois JM, et al (2006) Diagnostic performance of stress perfusion and delayed-enhancement MR imaging in patients with coronary artery disease. Radiology 240: 39–45. 16793971 10.1148/radiol.2401051161

[20] Gebker R, Jahnke C, Paetsch I, Kelle S, Schnackenburg B, et al (2008) Diagnostic performance of myocardial perfusion MR at 3 T in patients with coronary artery disease. Radiology 247: 57–63. 18305188 10.1148/radiol.2471070596

[21] Schwitter J, Nanz D, Kneifel S, Bertschinger K, Büchi M, et al (2001) Assessment of myocardial perfusion in coronary artery disease by magnetic resonance: a comparison with positron emission tomography and coronary angiography. Circulation 103: 2230–2235. 11342469 10.1161/01.cir.103.18.2230

[22] Thilo C, Schoepf UJ, Gordon L, Chiaramida S, Serguson J, et al (2009) Integrated assessment of coronary anatomy and myocardial perfusion using a retractable SPECT camera combined with 64-slice CT: initial experience. Eur Radiol 19: 845–856. 18972115 10.1007/s00330-008-1214-4

[23] Beller GA (2010) Importance of consideration of radiation doses from cardiac imaging procedures and risks of cancer. J Nucl Cardiol 17: 1–3. 20066526 10.1007/s12350-009-9189-3

[24] Kim RJ, Fieno DS, Parrish TB, Harris K, Chen EL, et al (1999) Relationship of MRI delayed contrast enhancement to irreversible injury, infarct age, and contractile function. Circulation 100: 1992–2002. 10556226 10.1161/01.cir.100.19.1992

[25] Gerber BL, Rousseau MF, Ahn SA, le Polain de Waroux JB, Pouleur AC, et al (2012) Prognostic value of myocardial viability by delayed-enhanced magnetic resonance in patients with coronary artery disease and low ejection fraction: impact of revascularization therapy. J Am Coll Cardiol 59: 825–835. 22361403 10.1016/j.jacc.2011.09.073

[26] Beek AM, Kühl HP, Bondarenko O, Twisk JW, Hofman MB, et al (2003) Delayed contrast-enhanced magnetic resonance imaging for the prediction of regional functional improvement after acute myocardial infarction. J Am Coll Cardiol 42: 895–901. 12957439 10.1016/s0735-1097(03)00835-0

